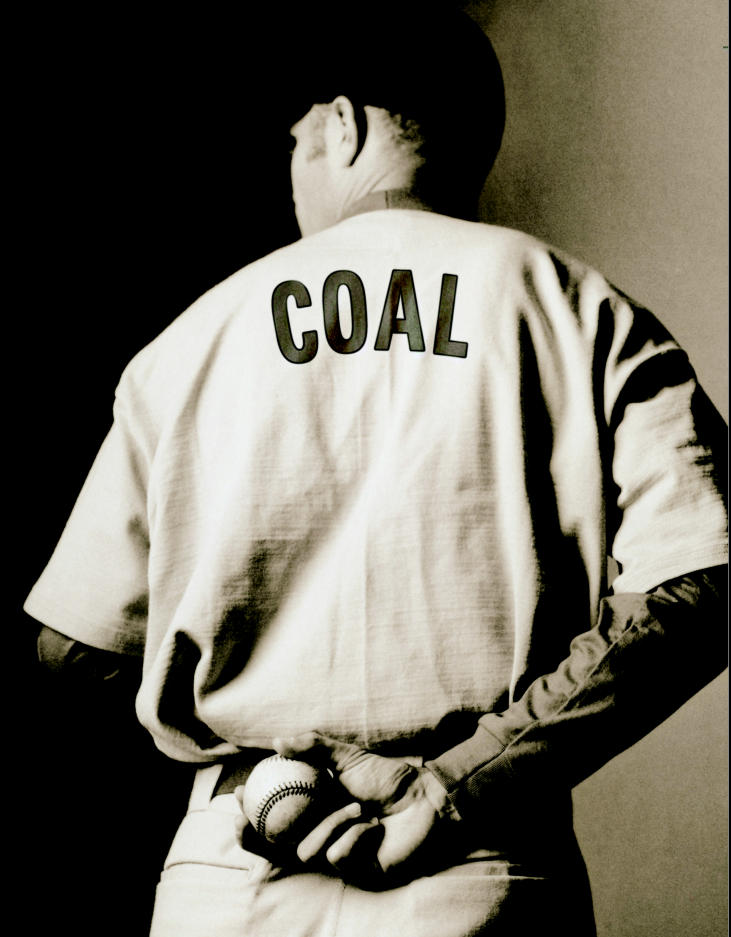# Coal Poised for a Comeback?

**DOI:** 10.1289/ehp.112-a888

**Published:** 2004-11

**Authors:** Scott Fields

Coal could be called energy’s comeback kid: sometimes forgotten, perhaps underappreciated, but always available for one more shot at the big time. It is one of humankind’s original sources of energy, and is used worldwide for cooking, heating, forging steel, and making electricity. In the United States, coal’s role today is limited almost exclusively to electricity generation; for the last decade or so, even that use has stagnated. For years new power facilities that relied on coal were spurned in favor of natural gas, as American electric companies were wooed by the cleaner-burning fossil fuel and its easier-to-site and cheaper-to-build power plants. Still, because so many coal-fired plants were built before the natural gas craze, coal accounts for over 50% of our annual electric generation. And now many energy experts say coal is poised to once again play a prominent role in the United States.

Coal does have an appeal. For one thing, there’s plenty of it. It’s located here in the United States, a comfort to those worried about the political and security hazards of overdependence on imported energy. It’s cheap. And its price is stable, at least compared to natural gas.

But coal can be ugly, too. If left unchecked with inadequate emissions control, it can emit ash (which has been linked to human cancers and genotoxic effects in some animal studies), sulfur dioxide (which contributes to acid rain), carbon dioxide (CO_2_; the chief culprit behind global warming), nitrogen oxides (NO_x_; which can produce smog and low-lying ozone), and mercury (linked to disorders in the kidneys and the nervous, digestive, and respiratory systems). Mining coal can also be a messy business, carving scars into the Earth, releasing clouds of dust, leaving behind sources of acidic water that can persist decades after a mine closes, and requiring dams—“impoundments” in industry lingo—that sometimes break and ravage miles of waterways.

In coming years, however, what’s right about coal will almost certainly overpower what’s wrong, says Richard Gendreau, a senior market consultant for R.W. Beck, a Framingham, Massachusetts, management consulting and engineering company. And what’s wrong, he says, will be made better by new technologies and more vigorous application of existing technologies. “The ultimate driver on all of this,” he says, “is that ninety-five percent of our fossil energy reserves—the amount of fossil energy that we have within our boundaries that we can rely on for energy and economic security, as well as national security—is coal.”

## The Saudi Arabia of Coal

Sometimes called the “Saudi Arabia of coal,” the United States has enough known reserves of coal—some 250–300 billion tons—to last at least 250 years, according to the Washington, D.C.–based National Mining Association. This coal, the association estimates, represents about one-quarter of the world’s known reserves and contains energy equivalent to all of the Earth’s known oil reserves. And that may be just the tip of the iceberg, says Connie Holmes, a senior economist and director of international policy for the association. If you consider sources that haven’t yet been discovered or are too impractical to extract, there is an estimated 2 trillion tons of coal in the United States. So if exploration and extraction technologies improve, there is a great deal more coal that could be recovered.

Although Gendreau and many other energy pundits predict a markedly growing role for coal in American electric production over the next two decades, current coal production is relatively stable, says Holmes. This year, she says, American coal mines will produce about 1.1 billion tons of coal. “That has been pretty much the case, give or take ten million tons, since 1996,” she explains. “There has not been an appreciable increase in production overall, but there has definitely been a shift in the market.”

In the past few years, U.S. steel producers have used far less coal, as foreign steel makers have surpassed American companies and domestic production has slacked off. Coal exports have also plummeted as cheap sources of coal have surfaced in Asia and South America. Holmes says these decreases have been offset by a resurgence in coal used to make electricity—coal use by utilities has gone up by about 120 million tons. In its *Annual Energy Outlook 2004*, the Energy Information Administration of the Department of Energy projects that the United States will use about 1.5% more coal each year between 2002 and 2025. But according to the National Mining Association, past estimates by this agency have fallen short. Predictions made in the 1990s for coal use in 2010, for example, were reached by 2000.

Although plentiful and available, coal had until recently fallen out of favor with U.S. utilities for new electric generation, Gendreau says. The deregulation of natural gas, partially in 1978 and then completely with the Natural Gas Wellhead Decontrol Act of 1989, drove down the cost of natural gas. Carter-era conservation programs, along with the completion of ongoing nuclear and coal projects, resulted in excess electric-generating capacity in most parts of the country throughout the 1980s and into the 1990s.

During this period, Holmes adds, utility managers were coasting on this excess capacity. They were uncertain about upcoming fuel markets and regulatory climates, and were steered by the Clinton administration, which encouraged natural gas use and discouraged coal use. As a result, through the 1980s and early 1990s there were few new coal power plants built as the nation’s thirst for electric power continued.

## The Advent of Natural Gas

“All of a sudden people realized in the early to mid nineties, ‘Oh my god, we’re running out of electric power,’” Gendreau says. At that time, natural gas was cheap, costing about $2–3 per million British thermal units (Btus). And new highly efficient gas turbine technologies allowed much greater power yields from burning gas.

These technologies made it possible for investment-shy utilities and the rapidly emerging nonutility generators—which produce power for the wholesale market—to build 500- to 750-megawatt modern, efficient, gas-powered electricity plants (by comparison, a modern coal-fired plant is typically in the 400- to 1,000-megawatt range, although larger units have been built). Not only were these plants less of an investment than a larger coal plant, they made it easier to meet environmental regulations because they naturally burn cleanly, without the controls required for coal plants. This also made them more attractive to citizens and environmental groups.

It was also much faster to build a gas-fired plant, Gendreau says. Just the permitting process for a coal plant takes two to three years, compared to about one year for a gas plant. Building a coal plant takes four to five years, compared to about two years for gas. Simply put, a natural gas plant does little more than send gas to a turbine; the gas is already in the form that is used. Coal, on the other hand, must be processed before use as a fuel, and there are side effects that must be managed. As a result, coal plants are more complicated. That’s also why they have to be larger—to take advantage of economies of scale.

Since the late 1990s, however, natural gas prices have doubled. “With natural gas prices over six dollars per million Btus, that makes coal much more attractive,” says Ned Helme, executive director of the Center for Clean Air Policy, a nonprofit organization in Washington, D.C. In fact, American utilities and nonutility generators have proposed constructing somewhere in the area of 100 new coal-fired plants, for an additional generating capacity of more than 57 gigawatts, according to the Department of Energy’s National Energy Technology Laboratory.

## Can Coal Really Cope?

But surrendering to coal’s skin-deep charms is the easy—and wrong—way out, says Jeff Deyette, an energy analyst for the Union of Concerned Scientists, a Cambridge, Massachusetts–based environmental organization. Even cleaner coal-fired plants aren’t clean enough, he says. Technologies to control coal’s by-products, especially CO_2_, are inadequate or unproven. And liberating coal always traumatizes the earth that surrounds it. A better approach, he says, would be to emphasize conservation and renewables, such as solar, biomass, geothermal, and wind. By 2020, Deyette says, renewables could deliver 20% of the nation’s electricity. The steps to this goal are described in the Union of Concerned Scientists’ 2001 report *Clean Energy Blueprint: A Smarter National Energy Policy for Today and the Future*.

“Left to their own devices, utilities will choose coal and natural gas because these are things that they’re comfortable with and because they don’t really have to account for all of the negative impacts of those fuels,” Deyette says. Although utilities must abide by emissions regulations, they don’t have to pay for the environmental costs of releasing NO_x_, ash, mercury, CO_2_, and other pollutants.

So how does one get the utilities to switch? “We’ve tried voluntary measures in the past,” Deyette says. “I think it’s time we move toward placing a requirement on utilities to increase renewables.” Currently 16 states—including Texas, Minnesota, and Wisconsin—have programs encouraging or requiring their utilities to invest in renewables.

Others counter that technical and political barriers prevent renewables from even keeping pace with the additional electricity the country requires each year, let alone making a dent in the total energy budget. Gendreau says, “There is no credible way to do this over such a short period. Even with incentives, renewables face many challenges and will remain a relatively small, but important and growing, part of our generation mix.”

Further, he says, it’s important that the cost impact to the rate payer be taken into consideration. “When dealing with such a critical component of our economy—as electricity certainly is—you can’t just impose such an extreme requirement, as laudable as it may be, and hope that somehow it happens,” he says.

## A Cleaner Coal Plant

Deyette says if the utilities are going to stick with coal, they should at least adopt more innovative technologies. But of the 100 or so proposed coal plants, only a couple deviate from the basic type—in which pulverized coal is burned while airborne in a furnace—that dominates the industry. “Almost all of the new coal plant proposals are, in fact, these older-generation technologies,” he says.

Others question this line of reasoning. “These technologies do represent advances as evidenced by the fact that emissions from coal utilities have been reduced by over thirty percent in the past two decades while electricity generated from coal has increased by approximately sixty-five percent,” says Paul Oakley, executive director of the Washington, D.C.–based Coalition for Affordable and Reliable Energy, an organization that represents the energy interests of companies and other organizations.

“Most of the plants . . . that are being proposed right now certainly utilize conventional power plant technologies, but it’s not necessarily grandma and grandpa’s technology,” Oakley says. “Just because they’re not new technologies doesn’t mean that they’re not advanced technologies. And they certainly emit fewer pollutants than the power plants we were seeing built twenty or twenty-five years ago.”

According to Gendreau, almost all modern power plants remove 99% or more of ash emissions and up to 95% of the sulfur, depending on the coal type and sulfur content. Power plants—new or old, although many older plants have been grandfathered to allow lower environmental standards—can be equipped with flue gas desulfurization equipment, commonly called “scrubbers,” which use a chemical reaction to convert sulfur dioxide from exhaust gas to a solid by-product. Plants can tame ash and other particulates with electrostatic precipitators or fabric filters. So-called selective catalytic reduction equipment can reduce NO_x_ emissions by exposing exhaust gas to a catalyst that triggers the NO_x_ to break down into nitrogen and water vapor. Utilities are testing newer technologies, such as activated carbon injection, to reduce mercury emissions.

But none of these methods reduce CO_2_ emissions. Gendreau says the only real method of reducing CO_2_ from coal burning is to burn the fuel more efficiently. That’s why “any new coal plant needs to be a coal gasification plant where the carbon can be captured and stored,” says Antonia Herzog, a legislative advocate for the Natural Resources Defense Council, a New York–based environmental organization. “The gasification plants are significantly better for even just conventional pollution.”

In coal gasification, solid coal is converted into a synthetic gas that is primarily carbon monoxide and hydrogen. Integrated gasification combined cycle (IGCC) technology is used to drive two types of turbines. The synthetic gas is combusted to drive gas turbines, which provide 60–70% of the power, and heat from the exhaust gas drives steam turbines, providing the rest of the power. Although IGCC units are somewhat cleaner in most respects when compared to a conventional pulverized-coal plant that is equipped with scrubbers, says Herbert Kosstrin, a senior director for R.W. Beck, where these units may excel is in capturing mercury and CO_2_ at less cost than in conventional plants.

## It’s All About the Money

In spite of these apparent advantages, full-blown IGCC plants are rare in the United States. And only a few of the proposed plants are proposed to be IGCC. “Until there are even more stringent regulations with emissions, you’re going to see conventional [plants built],” says Bruce Miller, director of the Pennsylvania State University Center for Fuel Utilization. “As the regulations become more stringent, you’ll see more gasification plants.”

As with most business decisions, it’s all about the money, Kosstrin says. As it becomes more expensive to pollute, as IGCC plants are proven to be more efficient, and as improved gasification systems are developed (the government is channeling hundreds of millions of dollars into clean-coal research, much of it centered on gasification), utilities will switch, he says.

That said, utilities are notoriously slow to accept new technologies, says J. Davitt McAteer, who was chief of the Department of Labor Mine Safety and Health Administration during the Clinton administration and who is now a special consultant on Appalachian affairs at Wheeling Jesuit University in West Virginia. “The industry continues in the old way to do business,” he says. “You’re talking about people who are constitutionally opposed to change and chance taking and ideas and concepts. It is going to take a sea change to shift both the utilities and the coal industry into a new mind-set.”

But unless this sea change is a reexamination of nuclear power or a massive change in the way Americans use energy, Gendreau says, the country is going to markedly increase its coal consumption. “When you look at all of the factors and consider all of the available options, you realize that no matter what you do, even as efficient as [Americans] are today, that you’re going to need more electricity—and there’s only one place to get it, and that’s coal,” he says.

“Coal will be part of the energy future of the United States,” Helme agrees, “but it’s critical that we address environmental issues now because we’re building that next fleet of power plants over the next twenty years. For the most part, we’re going to replace much of this by 2030, and what we replace it with is the whole game in terms of the climate issue.”

## Figures and Tables

**Figure:**